# The benefits of cholinergic enhancement during perceptual learning are long-lasting

**DOI:** 10.3389/fncom.2013.00066

**Published:** 2013-05-29

**Authors:** Ariel Rokem, Michael A. Silver

**Affiliations:** ^1^The Department of Psychology, Stanford UniversityStanford, CA, USA; ^2^School of Optometry, Helen Wills Neuroscience Institute, University of California, BerkeleyBerkeley, CA, USA

**Keywords:** acetylcholine, perceptual learning, vision, donepezil, neural plasticity

## Abstract

The neurotransmitter acetylcholine (ACh) regulates many aspects of cognition, including attention and memory. Previous research in animal models has shown that plasticity in sensory systems often depends on the behavioral relevance of a stimulus and/or task. However, experimentally increasing ACh release in the cortex can result in experience-dependent plasticity, even in the absence of behavioral relevance. In humans, the pharmacological enhancement of ACh transmission by administration of the cholinesterase inhibitor donepezil during performance of a perceptual task increases the magnitude of perceptual learning (PL) and its specificity to physical parameters of the stimuli used for training. Behavioral effects of PL have previously been shown to persist for many months. In the present study, we tested whether enhancement of PL by donepezil is also long-lasting. Healthy human subjects were trained on a motion direction discrimination task during cholinergic enhancement, and follow-up testing was performed 5–15 months after the end of training and without additional drug administration. Increases in performance associated with training under donepezil were evident in follow-up retesting, indicating that cholinergic enhancement has beneficial long-term effects on PL. These findings suggest that cholinergic enhancement of training procedures used to treat clinical disorders should improve long-term outcomes of these procedures.

## Introduction

**Figure 1:**
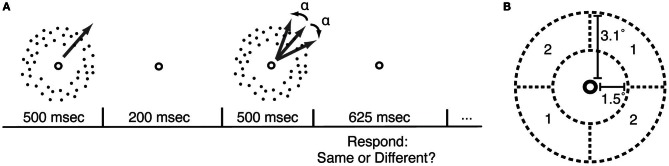
**Motion direction discrimination (MDD) task. (A)** In each trial, participants viewed two sequentially-presented fields of dots that were moving in either the same or different directions. MDD thresholds were measured by applying a psychophysical staircase (Watson and Pelli, 1983) to the angular difference in directions between the two motion stimuli (α). **(B)** During each course of PL, participants practiced the MDD task for one location (quadrant pair 1 or 2) and one direction of motion.

Perceptual learning (PL) is the improvement of performance on a perceptual task with training. One of the defining characteristics of PL is its specificity to the physical parameters of the stimuli used for training (Sagi, 2011). For example, when participants practice a motion direction discrimination (MDD) task (Figure 1A) for a particular direction of motion and in a particular location in the visual field, the resulting improvement in performance does not fully generalize to other directions of motion or to other visual field locations (Ball and Sekuler, 1982, 1987). Another hallmark of PL is the persistence of learning for extended periods of time. For example, the effects of PL on MDD can be observed several months after the training procedure has ended (Ball and Sekuler, 1982, 1987).

The specificity of PL to the training stimulus has led to the hypothesis that PL occurs via long-term changes in the responses of neurons in early visual cortex (e.g., Karni and Sagi, 1991). However, others have argued that specific learning could be the result of changes in the decoding process, rather than in stimulus encoding (Mollon and Danilova, 1996; Dosher and Lu, 1999). This is consistent with recent experiments demonstrating that specificity of learning is reduced by training on a different task in another location (Xiao et al., 2008), conducting a brief perceptual test in another location before training (Zhang et al., 2010a) or passive exposure to stimuli other than the trained stimulus (Zhang et al., 2010b). Nevertheless, changes in neural selectivity in visual cortex have been described in animal models following PL (Schoups et al., 2001; Yang and Maunsell, 2004). In some cases, cortical plasticity has been shown to depend on the relevance of the training stimulus to the task performed by the animal. For example, cortical reorganization in adult animals only occurred when the stimulus was behaviorally relevant and not when animals were passively exposed to the stimulus (Recanzone et al., 1993).

The neurotransmitter acetylcholine (ACh) is involved in the regulation of many cognitive functions, including attention and learning (Hasselmo and Sarter, 2011). ACh is synthesized by neurons in the basal forebrain and is released by the axons of these neurons throughout the cerebral cortex. The role of ACh in facilitating plasticity has been demonstrated in animal models by increasing ACh signaling in cortex while animals were passively exposed to a stimulus. Repeated pairing of visual stimulus presentation and local infusion of ACh selectively increased responses of V1 cells to the stimulus that had been paired with ACh (Greuel et al., 1988). Similarly, in rodents, pairing of electrical stimulation of the basal forebrain and auditory stimulation with a tone at a particular frequency resulted in reorganization of the primary auditory cortical map such that the size of the auditory cortical region that responded to the previously paired tone increased (Kilgard and Merzenich, 1998). Both of these findings suggest that synchronous ACh signaling and stimulus presentation modified the receptive fields of neurons in a stimulus-specific manner. These findings are consistent with the proposal that ACh release during task performance regulates sensory cortical plasticity by shifting the responses of populations of cortical neurons toward encoding of extrinsic inputs and away from intrinsic feedback signals involved in recall (Giocomo and Hasselmo, 2007).

Cholinesterase inhibitors are a class of drugs that raise the levels of ACh in the synapse by inhibiting the activity of the cholinesterase enzyme that metabolizes ACh. These drugs are commonly prescribed for the treatment of Alzheimer's disease, a condition characterized by loss of cortical cholinergic tone (Francis et al., 2010). In a previous study (Rokem and Silver, 2010), we demonstrated that enhancement of cholinergic transmission by the cholinesterase inhibitor donepezil (trade name: Aricept) increased the magnitude and specificity of visual PL of motion direction discrimination (MDD; Figure 1A) in healthy humans. The long-term maintenance of PL effects is particularly important in cases in which PL is used to treat clinical conditions, such as amblyopia (Levi and Li, 2009). Pharmacologically enhanced PL procedures would be most beneficial for these conditions if these procedures did not require continuous additional administration of the drug after the end of training. In the present study, we tested whether the effects of donepezil on PL were maintained several months after the end of drug administration and training.

## Materials and methods

### Subjects

Eight of the 12 original participants in the study described in Rokem and Silver (2010) participated in follow-up testing. All participants had normal or corrected-to-normal vision (4 female; mean age: 22.8 years, SD: 7.2). Participants provided informed consent and were monetarily compensated for their participation. The study procedures were approved by the Committee for the Protection of Human Subjects at UC Berkeley.

### Procedure

To assess the long-term effects of cholinergic enhancement during PL, we measured MDD thresholds for two different locations in the visual field (Figure 1B) and for 8 directions of motion in each location. Participants returned to the laboratory for retesting 5–15 months after the end of training. As in the original study, subjects were seated 150 cm from a NEC Multisync FE992 CRT monitor, and their heads were stabilized with a chin rest. As in our previous studies on MDD (Rokem and Silver, 2009, 2010), the edges and corners of the screen were covered with a circular black aperture to prevent subjects from using them as cues for the MDD task.

### Stimulus

Random dot kinetograms (RDK) were identical to those described in our previous study (Rokem and Silver, 2010). The RDKs were presented within a circular annulus covering 1.5–3.1° radius from the fixation point. Two quadrants of the annulus, located on opposite sides of the fixation point (Figure 1B), contained 100% coherent motion. The remaining quadrants contained 0% coherent motion. Dots moved to a new position within the annulus after two monitor refresh frames in order to prevent the possibility of judging motion direction by tracking a single dot. Dots were 1.8 arcminutes in size, dot density was approximately 8.5 dots/degree^2^, and dot velocity was 8°/second. Stimuli were created using the Psychophysics Toolbox (Brainard, 1997; Pelli, 1997) for Matlab. In each trial, two RDK stimuli were presented sequentially (Figure 1A), and subjects reported whether the stimuli in the two intervals were moving in the same direction or in different directions. The angular difference between the stimuli in the 50% of trials in which the stimuli were moving in different directions was adjusted using a QUEST psychophysical staircase (Watson and Pelli, 1983), and thresholds for 70% correct performance were determined from all the trials in each staircase. Software implementing this task is available at: https://github.com/arokem/motion_th

### Analysis

In the original study (Rokem and Silver, 2010), we used a placebo-controlled crossover design. Half of the participants (the “donepezil first” group) initially underwent a training procedure while taking donepezil, followed by a second training procedure conducted while taking placebo. The other half of the participants (the “donepezil second” group) trained first under placebo, followed by a second course of training under donepezil. Of the original 12 participants, only 8 were available to participate in the current follow-up study: three of these were in the “donepezil first” group and five were in the “donepezil second” group.

MDD thresholds in the eight different directions and two different visual field locations were analyzed at three different times: the very first pre-training measurement (conducted under donepezil for the “donepezil first” group and under placebo for the “donepezil second” group), the second post-training session (approximately one day after completion of the second course of training), and in the follow-up assessment. These thresholds were analyzed using a mixed-model ANOVA, with visual field location (location trained under donepezil or under placebo), direction of motion (relative to the direction trained in that location), and time point (first pre-training, second post-training, or follow-up assessment) as within-subject factors and training group (“donepezil first” or “donepezil second”) as a between-subject factor.

For each participant, we also calculated percent learning relative to *threshold (original)* (the threshold obtained in the first pre-training measurement):

$$100\cdot \left[1-\frac{\text{threshold}(\text{current})}{\text{threshold}(\text{original})}\right]$$

For each participant, percent learning values were computed for the condition (combination of motion direction and visual field location, see Figure 1) that was trained while the participant was taking donepezil (“donepezil condition”), for the condition that was trained while the participant was taking placebo (“placebo condition”), and for all other direction/location combinations that were not trained in either of these (“untrained conditions”).

We conducted a Two-Way ANOVA on the percent learning scores, with training condition (“donepezil condition,” “placebo condition,” and “untrained conditions”) as a within-subject factor and training group (“donepezil first” or “donepezil second”) as a between-subject factor. In addition, differences in thresholds and differences in percent learning between conditions were directly assessed using a signed-rank test.

## Results

In our original study (Rokem and Silver, 2010), each participant underwent two sequential courses of MDD training. In one course of training, 5 mg of donepezil was administered daily throughout pre-training measurements, training, and post-training measurements. In the other course of training, an identical procedure was used, except subjects ingested an inactive placebo every day. The order of drug/placebo administration in the two courses of training was counterbalanced between subjects, and drug/placebo administration was double-blind.

Before and after each course of training, participants were tested in 8 different directions of motion and two different locations in the visual field (Figure 1B). For the first course of training (donepezil or placebo), one of these direction/location combinations was designated as the trained condition. For each subject, the opposite direction and the other location were the trained condition in the second course of training. Because PL of MDD is specific for stimulus direction and location (Ball and Sekuler, 1987), this experimental design allowed us to separately measure effects of training under placebo and under donepezil for each subject.

Measurement of the effects of PL one day after the end of each course of training (while subjects were still receiving donepezil or placebo) showed that training under donepezil resulted in greater PL than training under placebo (Rokem and Silver, 2010). Moreover, the effects of training were more specific under donepezil: there was less generalization of improvement in performance to untrained locations and to untrained directions under donepezil than under placebo (Rokem and Silver, 2010).

To measure long-term retention of the effects of donepezil on PL in the current study, we tested 8 of the original 12 participants in a follow-up experiment 5–15 months after the end of the original study. We measured MDD thresholds for all combinations of the two spatial locations (Figure 1B) and the eight directions used in the original study. To assess learning, we obtained thresholds (2 locations × 8 directions) for each participant at three different times: (1) in the initial measurements that we collected from each subject before any training had occurred, (2) one day after completion of the second course of training, and (3) in the follow-up testing session. Note that for three of the participants (the “donepezil first” group), the initial measurement was obtained while they were taking donepezil, and the post-training measurement was obtained under placebo. For the other five participants (the “donepezil second” group), the initial measurement was obtained under placebo, and the post-training measurement was obtained under donepezil.

**Figure 2:**
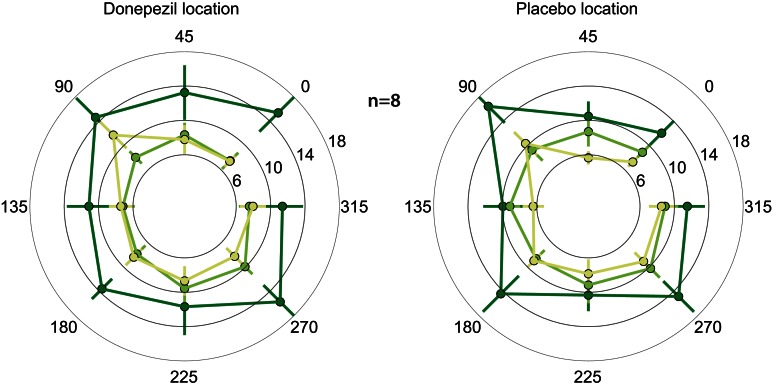
**Motion direction discrimination thresholds.** Thresholds for each combination of location and direction of motion were assessed at three different time points: before any training (dark green), one day after the completion of the second course of training (light green), and 5–15 months after training (yellow). Thresholds were separately averaged across subjects for the location that was trained under donepezil (left) or under placebo (right). In each location, the trained direction is defined as zero degrees, and all other directions are rotated accordingly. Thresholds decreased following training (light green < dark green), and there is no evidence of decay in the benefits of training in follow-up testing (light green similar to yellow).

In both the location trained under donepezil and the location trained under placebo, thresholds in the trained direction, as well as the other directions, decreased substantially over the course of training (Figure 2; *F*_(1, 364)_ = 9.2, *p* = 0.003). Importantly, thresholds in follow-up testing are almost identical to the immediate post-training thresholds in almost all conditions. In particular, thresholds in the donepezil-trained condition are virtually identical for post-training (7.4 ± 1.1°) and follow-up testing (7.4 ± 0.7°). Similar results were obtained for the threshold in the placebo-trained condition: 8.9 ± 1.1° in the immediate post-training assessment and 7.3 ± 0.6° in follow-up testing. In conclusion, we find no evidence for decay of learning between the end of training and follow-up testing several months later.

There was also a time of testing-by-group interaction [*F*_(1, 364)_ = 4.3, *p* = 0.037] that was driven by a difference in the pre-training threshold between the donepezil-trained and the placebo-trained conditions. This difference approaches statistical significance (rank test, *p* = 0.05), but it is mainly due to one participant who had a much higher threshold in the donepezil pre-training condition than the rest of the participants (*z*-score = 2.42). When this subject's data were excluded, the difference between donepezil and placebo pre-training conditions was no longer significant (rank test, *p* = 0.1). Importantly, this participant's data do not account for any of the conclusions we present below.

The mean threshold in the untrained conditions at the time of follow-up testing was 8.3 ± 1.1°, and there was no significant effect of the different conditions (placebo-trained, donepezil-trained, and untrained) on raw threshold values at this time point. However, post-training raw thresholds are not the best measure of learning, because they contain both between-subject and within-subject (across locations and directions of motion) variability in performance prior to training. We therefore computed percent learning scores for each subject (relative to that subject's initial pre-training thresholds) for the direction/location combination that was trained under donepezil (“donepezil condition”), trained under placebo (“placebo condition”), and the average of all direction/location combinations that were not trained in either course of training (“untrained conditions”). Percent learning was larger for the donepezil condition (47.1 ± 4.6) than both the placebo condition (34.2 ± 6.9) and the untrained conditions (26.5 ± 4.0). Moreover, there was a significant effect of training condition (donepezil/placebo/untrained) on percent learning [*F*_(2, 12)_ = 6.0, *p* = 0.016], but there was no significant effect of training group (“donepezil first” vs. “donepezil second”) and no significant interaction of the two factors.

**Figure 3:**
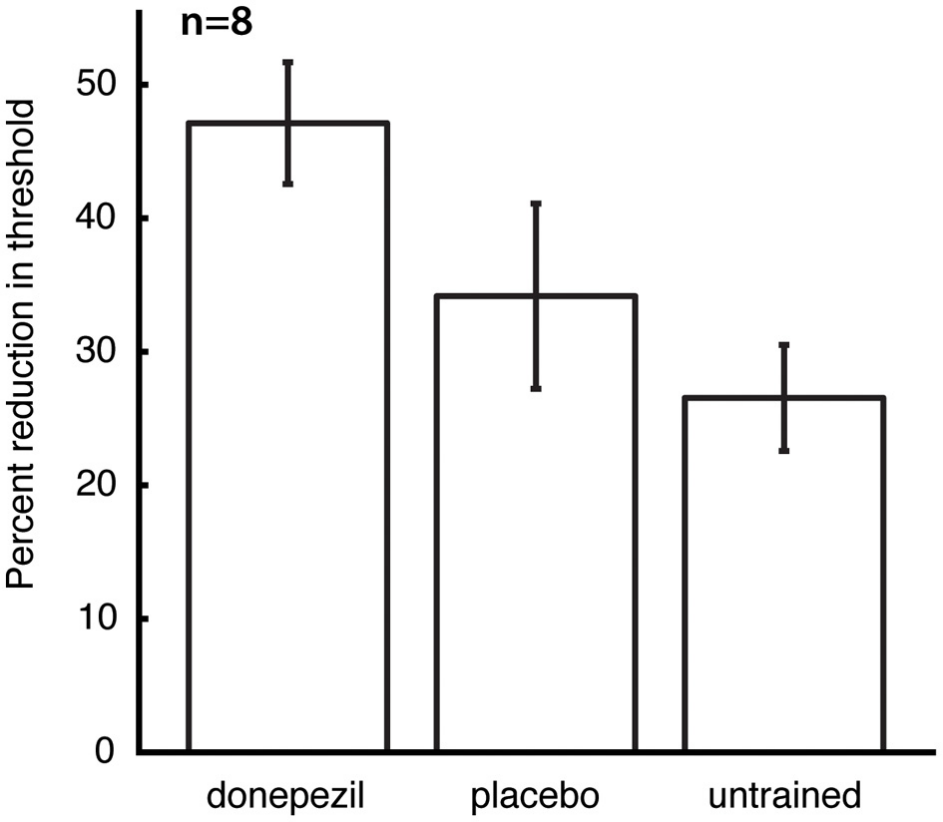
**Long-term retention of the benefits of training.** For each subject, percent learning was computed for each training condition (donepezil, placebo, and the mean of location/direction combinations that were not trained under either), relative to the initial pre-training measurement for that direction/location combination. Error bars are standard errors of the mean within each condition.

**Figure 4:**
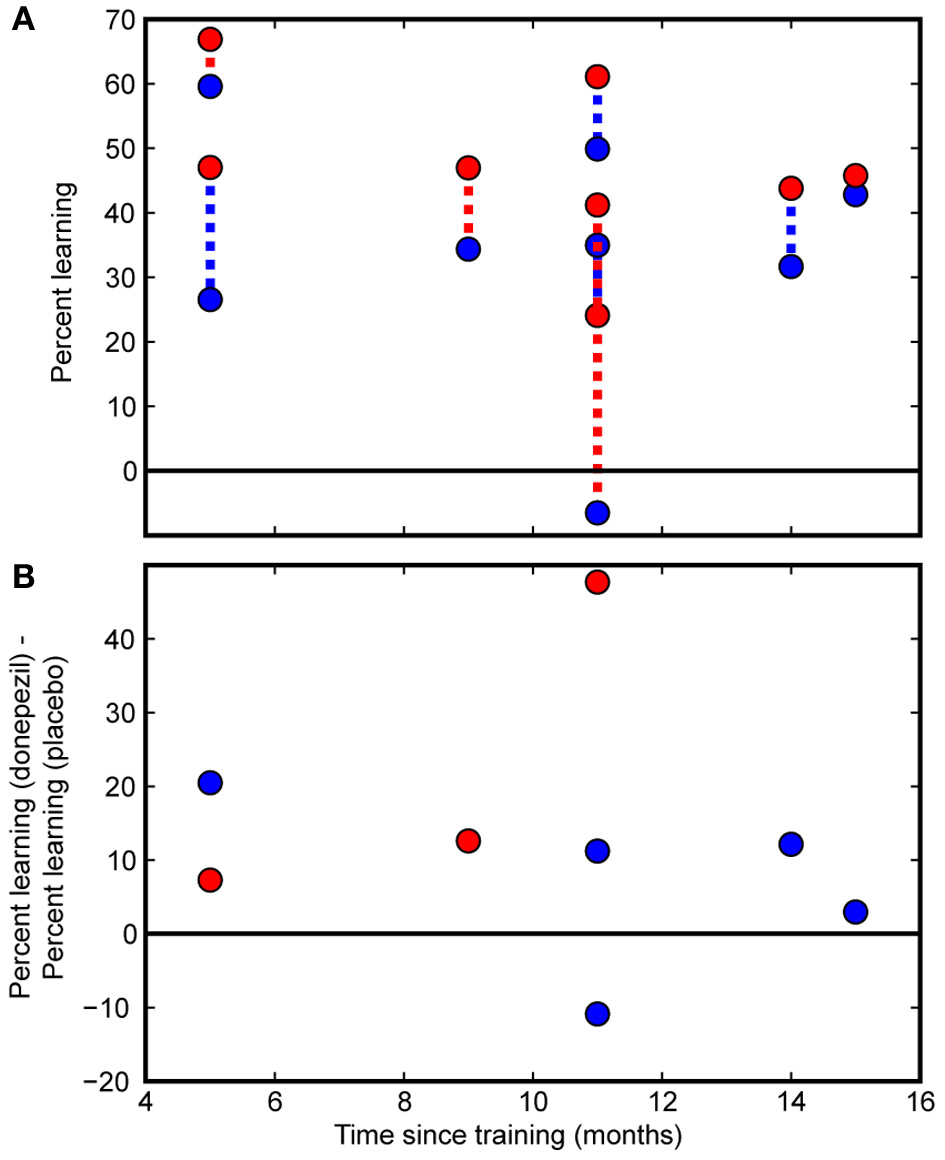
**Individual subject data.** Individual participants' data are presented as a function of the time interval between the initial course of training and follow-up measurements. **(A)** Percent learning for each subject in the donepezil-trained condition (filled red circles) and placebo-trained condition (filled blue circles). The two percent learning scores for each subject are connected with a dashed line. For participants in the “donepezil first” group, the dashed line is red. For the “donepezil second” subjects, the dashed line is blue. There was no indication of decay of learning following the cessation of training. **(B)** Within-subject differences between the donepezil-trained and the placebo-trained conditions. Percent learning was greater for the donepezil-trained condition than the placebo-trained condition in 7 out of 8 participants.

Direct comparisons revealed that there was significantly more long-lasting learning in the condition trained under donepezil than in the condition trained under placebo (signed-rank test, *p* = 0.036) (Figure 3) as well as more learning in the condition trained under donepezil compared to the average of the untrained conditions (signed-rank test, *p* = 0.012) (Figure 3). Numerically, 7 of 8 participants exhibited more learning in the condition trained under donepezil than in the condition trained under placebo (Figure 4). An alternative measure of PL is the difference in MDD threshold before and after training, computed for each subject. The average of this measure was also significantly larger in the condition trained under donepezil than in the condition trained under placebo (signed-rank test, *p* = 0.036).

Finally, we tested whether PL gradually decayed without additional exposure to the stimulus or additional cholinergic enhancement after the end of training. Even though our sample of 8 subjects spanned a large range of intervals between initial training and the follow-up testing procedure (5–15 months), there was no detectable effect of the duration of this interval on percent learning. Specifically, there were no significant correlations between number of months since the beginning of training and any measure of PL (% learning for condition trained under donepezil: *r* = −0.40, *p* = 0.3; % learning for condition trained under placebo: *r* = −0.15, *p* = 0.7; difference between donepezil and placebo: *r* = −0.12, *p* = 0.8; Figure 4).

## Discussion

We found that the beneficial effects of pharmacological cholinergic enhancement on PL are long-lasting. Previous work has shown that PL of the MDD task is maintained for at least several months following training (Ball and Sekuler, 1982, 1987), and our previous study (Rokem and Silver, 2010) demonstrated that PL of this task is augmented by cholinergic enhancement with donepezil. However, it was not clear from that study whether the augmentation of PL by donepezil would extend beyond the time at which the drug was eliminated from the bodies of the participants. In the present study, we found that pharmacological enhancement of the cholinergic system during PL has long-term benefits for task performance. Specifically, the additional improvement in MDD for the stimuli trained under donepezil is maintained for many months after donepezil administration and training have ended, and this long-lasting improvement is greater than the corresponding PL-induced improvement resulting from training under placebo. Moreover, we found no evidence for decay of the beneficial effects of training under donepezil over the 5–15 month time period we studied here.

It is unlikely that our findings are due to state-dependent learning, in which retrieval of learned information is facilitated if the organism is in the same physiological and/or psychological state as it was during learning (Godden and Baddeley, 1975). For example, pre-training injection of ethanol causes an amnesic effect if subsequent testing is conducted without additional administration of ethanol, but if ethanol is administered again prior to testing, complete recall is achieved (Nakagawa and Iwasaki, 1995). A cellular analog of state-dependent learning involving cholinergic transmission has also been reported, in which simultaneous infusion of ACh in rat somatosensory “barrel” cortex and vibrotactile whisker stimulation induced changes in neuronal tuning that continued to be expressed only if additional ACh was subsequently infused into cortex (Shulz et al., 2000). If state-dependent learning had occurred in our study, differences between training conditions would have been observed only if additional cholinergic enhancement occurred during the follow-up testing session.

What are the biological mechanisms of cholinergic enhancement of PL? One possibility is that ACh augments plasticity by increasing the gain of populations of cortical neurons that enable performance of the task. Attention is thought to play an important role in PL (Ahissar and Hochstein, 1993). Even in cases in which learning occurs when attention is directed away from the stimulus (Watanabe et al., 2001), the timing of attention allocation seems to play a role in facilitating learning (Seitz and Watanabe, 2003; see also Roelfsema et al., 2010 for a review of these issues). ACh is critically involved in the allocation of attention. In rodents, cortical ACh release increases during the performance of attentionally-demanding tasks (Arnold et al., 2002), and basal forebrain lesions impair the performance of attentional tasks (Muir et al., 1994). In addition, iontophoretic infusion of ACh in macaque primary visual cortex increases attentional modulation of neural responses, and this attentional modulation is mediated by muscarinic ACh receptors (Herrero et al., 2008). In humans, donepezil increases the beneficial effects of voluntary attention on visual performance (Rokem et al., 2010). Therefore, one possible explanation of the cholinergic effects on PL is that donepezil enhances attention during training, thereby increasing the facilitatory effects of attention on PL. The role of ACh and other neuromodulatory systems in enabling PL has been discussed in recent reviews (Seitz and Dinse, 2007; Seitz and Watanabe, 2009).

However, it is also possible that ACh exerts its effects on PL through post-training memory consolidation. Some studies have found that consolidation of PL does not occur until approximately 4–6 h following training (Karni and Sagi, 1993), and others have shown that sleep plays an important role in PL consolidation (Stickgold et al., 2000) and that donepezil increases the duration of REM sleep (Nissen et al., 2006). A recent study has replicated our finding of increased PL with enhanced cholinergic transmission, using a different task (texture discrimination) and with a different pharmacological manipulation (stimulation of nicotinic ACh receptors by chewing tobacco) (Beer et al., 2012). In this study, chewing tobacco was administered immediately after the end of the training procedure in order to distinguish the effects of nicotine on PL consolidation from nicotinic effects during training. Therefore, the results of Beer et al. (2012) suggest that nicotinic ACh receptors play a facilitatory role in post-training consolidation of PL. Our results are consistent with an action of donepezil during consolidation, during task performance, or through a combination of these mechanisms, and resolving the relative contributions of cholinergic effects on training and consolidation of PL is an important goal for future research.

Regardless of the mechanism of cholinergic facilitation of PL, our finding that this facilitation lasts for several months after the end of training and donepezil administration has important implications for cases in which PL is used to treat clinical conditions (Levi and Li, 2009). For example, PL improves visual acuity in patients with amblyopia immediately after training, and there is substantial retention of this improvement 12 months after the end of training (Polat et al., 2004). Similarly, enhancement of contrast sensitivity and visual acuity in patients with amblyopia is evident 18 months after training on a contrast detection task (Zhou et al., 2006). For cholinergic enhancement to benefit clinical applications of PL procedures, it is important that the pharmacological augmentation of learning persists well after the completion of training and drug administration. Our results suggest that the long-term benefits conferred by PL in the treatment of clinical disorders of vision may be augmented by pharmacological cholinergic enhancement during training without the need for long-term administration of the drug.

### Conflict of interest statement

The authors declare that the research was conducted in the absence of any commercial or financial relationships that could be construed as a potential conflict of interest.
